# Allopurinol-Induced Oral Lichenoid Drug Reaction with Complete Regression after Drug Withdrawal

**DOI:** 10.3390/dermatopathology7010004

**Published:** 2020-08-12

**Authors:** Alexandre Perez, Benjamin Lazzarotto, Jean-Pierre Carrel, Tommaso Lombardi

**Affiliations:** Unit of Oral Medicine and Pathology, Division of Oral Maxillofacial Surgery, Department of Surgery, University Hospitals of Geneva, 1205 Geneva, Switzerland; alexandre.perez@hcuge.ch (A.P.); benjamin.lazzarotto@hcuge.ch (B.L.); jean-pierre.carrel@unige.ch (J.-P.C.)

**Keywords:** oral mucosa, lichen planus, lichenoid reaction, adverse drug reaction, allopurinol

## Abstract

*Background*: Lichen planus is a chronic mucocutaneous inflammatory disease. Oral manifestations are common, and may remain exclusive to the oral mucosa without involvement of the skin or other mucosae. A differential diagnosis includes oral lichenoid drug reactions. Allopurinol, which is the first line hypo-uricemic treatment, is often quoted as being a possible offending drug, though oral reactions have rarely been reported. *Case presentation*: We describe a 59-year-old male gout patient, successfully treated with allopurinol, who developed acute onset of oral lichenoid lesions, involving bilaterally the buccal mucosa, the tongue and the labial mucosa. Histopathology was consistent with a lichen planus or a drug-induced lichenoid reaction. Improvement of the patient’s condition after withdrawal of allopurinol confirmed the lichenoid nature of the lesion. Remission was complete after a few weeks. *Discussion*: Although unusual, allopurinol may induce a lichenoid drug reaction. These reactions may mimic clinically and histopathologically idiopathic lichen planus. Improvement or complete regression of the lesions may be attempted to confirm the diagnosis. According to the latest WHO recommendations, these lesions have a potential for malignant transformation.

## 1. Introduction

Lichen planus is a common chronic inflammatory disorder, affecting the skin, oral and genital mucosa, scalp and nails. Oral manifestations are frequent, and may remain exclusive to the oral cavity without involvement of other organs [1].

To the contrary of cutaneous lichen planus, oral lichen planus is a long-term chronic disease with a dynamic evolution, as a result of successive waves of variably destructive activity at the epithelium–chorion interface. Thus, progressive changes of the clinical and histopathological aspects occur over time, increasing the number of possible clinical presentations, going from white keratotic dots to mucosal atrophy and hyperkeratosis in the late stage. The most characteristic and easily recognized clinical aspect being the reticular form [2].

Oral lichenoid drug reactions are a connected entity, the term referring to a lichen planus-like rash triggered by systemic drug exposure. Allopurinol stands beside many classes of drugs involved, such as nonsteroidal anti-inflammatory drugs (NSAIDs), B-blockers, ACE inhibitors, thiazide diuretics as well as some antibiotics [3]. This anti-gout drug is often quoted but only a few reports are described in the literature.

This article reports a clinically and histopathologically detailed case of oral lichenoid lesions associated with allopurinol therapy, that showed complete regression after the withdrawal of the drug.

## 2. Case Presentation

**Figure 1:**
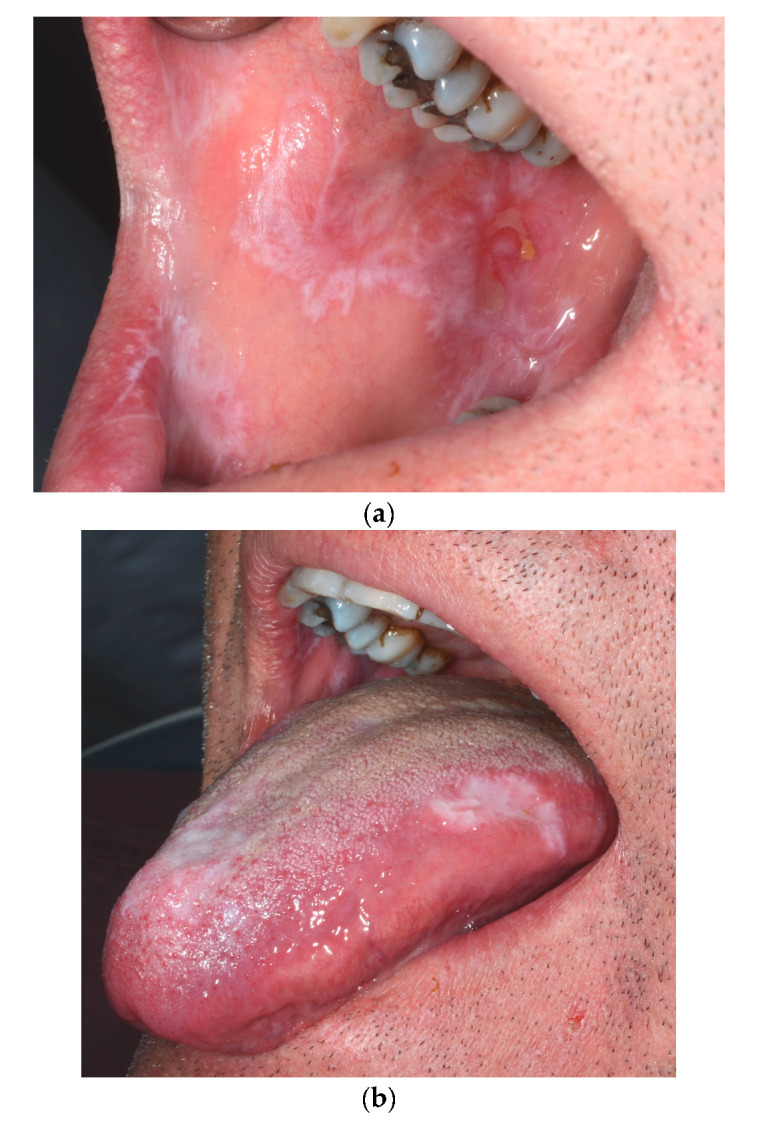

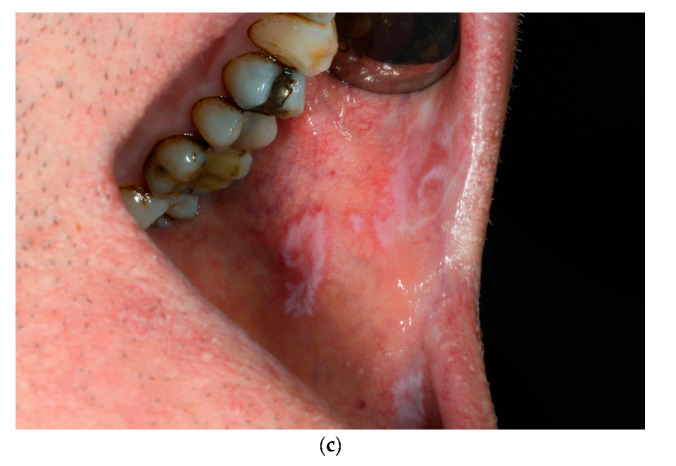
(**a**) Keratotic lesions involving the right buccal mucosa and the lower lip, with focal erythema and an ulcerated area. (**b**) Keratotic lesions involving the lateral border of the tongue, and the median anterior area. (**c**) Keratotic lesions of the left buccal mucosa and lip.

A 59-year-old man was referred due to a 4-week history of severe pain within the oral cavity. The patient give written consent for publication. Medical history revealed skin psoriasis diagnosed with a biopsy more than 30 years ago, which has since been treated by calcipotriol betamethasone gel (Daivobet^®^, LEO Pharmaceutical Products Ltd., Regensdorf, Switzerland) intermittently depending on the evolution of the lesions. Otherwise, the patient suffered episodic gout attacks for which he was under allopurinol for one month prior to consultation. On examination, he had multiple keratinized lesions in plaque form, involving the bilateral buccal and lingual mucosae, with some erythematous streaks and ulcerated areas (Figure 1).

**Figure 2:**
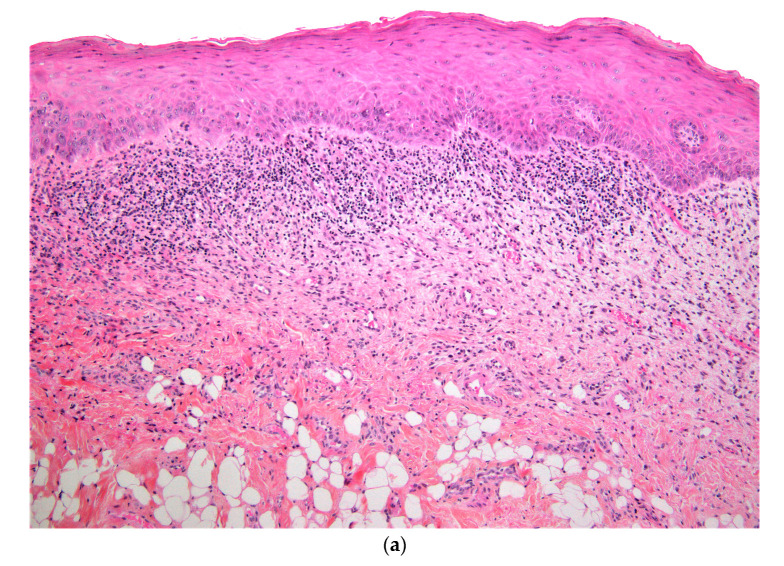

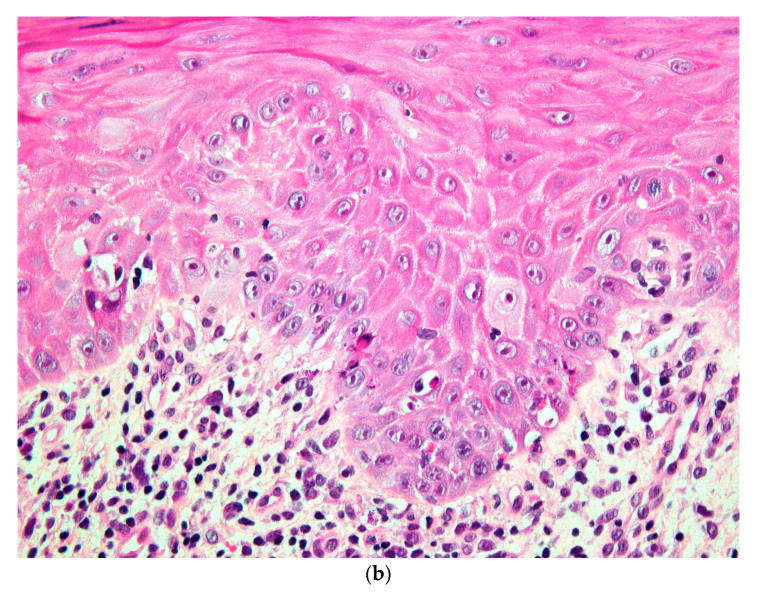
(**a**) Histopathological section showing parakeratotic partly atrophic squamous epithelium with a band-like dense inflammatory infiltrate in the superficial chorion (HE stain, ×10). (**b**) Higher magnification showing inflammatory lymphocytic infiltrate and apoptotic bodies in the basal layer (HE stain, ×40).

A biopsy of the right buccal mucosa was performed. Histopathological examination revealed a stratified squamous epithelium with focal atrophy and discreet parakeratosis. The superficial chorion contained a band-like dense inflammatory infiltrate, composed mostly of lymphocytes and macrophages, with very rare eosinophils. Apoptotic keratinocytes in the basal layer were observed (Figure 2a,b). These features were consistent with an active lichen planus or a drug-induced lichenoid reaction.

**Figure 3:**
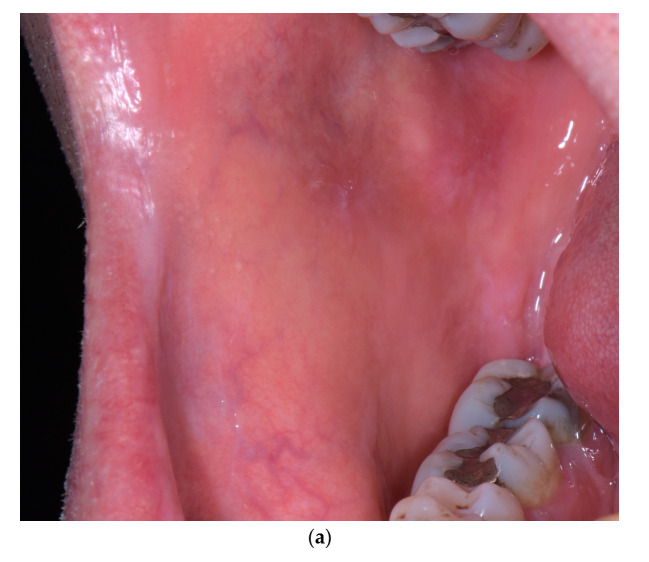

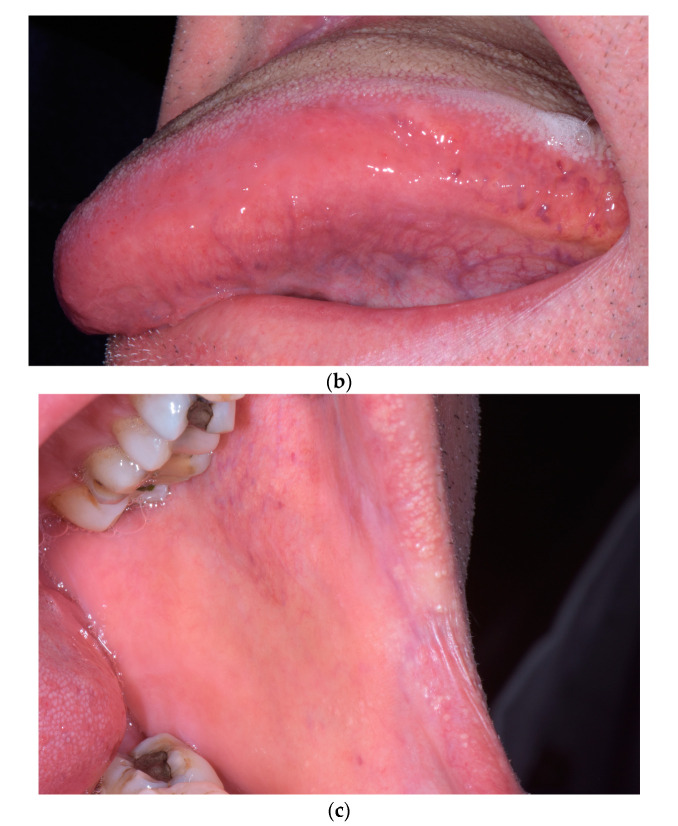
(**a–c**) Three-year follow-up showing complete healing of the right buccal mucosa and the lower lip (**a**), the lateral border of the tongue (**b**), and the left buccal mucosa and lip (**c**).

Given the suspected allopurinol involvement, the medication was withdrawn and not substituted in agreement with his general practitioner. Three weeks after discontinuation, significant regression of the lesions was observed and the patient no longer had any symptoms. The one-year postoperative examination revealed a thin keratosis of the buccal mucosa and a discreet depapillation of the dorsolingual mucosa. Follow-up after 3 years showed complete healing (Figure 3).

## 3. Discussion

Gout is the most common form of arthritis in men over the age of 40 and its incidence keeps on increasing [4,5]. Allopurinol is an effective urate-lowering drug, working as a xanthine oxidase inhibitor. It is considered as the first line preventative treatment of chronic gout worldwide. Some infrequent adverse events are well described, mainly the hypersensitivity syndrome. Skin reactions may occur in approximately 2% of patients. Most cutaneous reactions are maculo-papular eruptions; however, severe reactions, such as toxic epidermal necrolysis, are also documented [6].

Oral manifestations in the form of lichenoid lesions, although frequently quoted, are rare, and have only been reported twice in the literature [7,8] (see Table 1). The clinical presentation in all reported cases was painful oral ulcerations. Patients were 1 female and 3 males, and patient age ranged from 53 to 75 years. Lesions were described as white striae, white plaques, along with erosions and ulcerations. All lesions were present bilaterally on the buccal mucosa, on the borders of the tongue, and in two cases on the mucosa of the lower lip. Clinical impression, in all reported cases, was that of erosive lichen planus. A biopsy was performed in only one case [7]. The latter was said to be consistent with lichen planus without further details.

In our case, the patient complained of pain and the buccal mucosa and tongue were affected, with erosions on the former. Histologically, our case showed a lichenoid aspect although indistinguishable from an idiopathic lichen planus. The presence of parakeratosis is not helpful in oral lesions and eosinophils were very rare, differently to drug-induced skin lesions.

The diagnosis of drug-induced lichenoid lesions may be difficult, as it may share a similar aspect to those found in the idiopathic oral lichen planus, both clinically and histopathologically. It is important to make the difference between the two conditions, because lichen planus is usually treated by corticosteroids or immunomodulatory agents, whereas drug-induced lichenoid lesions are treated by the withdrawal of the offending agent.

The difficulty of diagnosis also lies in the fact that cutaneous and/or oral lesions may occur after a variable latency period from the introduction of the offending drug [3]. In the present case, a few weeks of allopurinol therapy were sufficient to observe oral lesions, which is consistent with the two previous reports.

Our diagnosis was conducted according to the updated French drug reaction assessment of imputability criteria [9]. In the present case, the chronological score was 3 (C3) whereas the semiological score was 2 (S2). Therefore, intrinsic imputability score was 5 (I5), and extrinsic imputability score was 2 (B2) considering the bibliographical data. Imputability of allopurinol on the basis of the obtained score was consistent.

The resolution after drug withdrawal seems to be spread over a variable period of time, and the literature is evasive about the degree of regression that the clinician can expect. In the present case, complete healing was observed at the 3-year follow-up. For other drugs, some authors suggest a time frame of up to 24 months before full resolution, depending on the initial extension of the lesions, their severity or the drug incriminated [3,10].

Although the World Health Organization (WHO) classifies oral lichen planus as a precancerous disease, incidence of malignant transformation is still a matter of discussion. Discrepancies in studies may stem from variations in diagnostic criteria, insufficient knowledge or recognition of the late stage of the disease (post-lichen state), confusion with other keratotic and/or atrophic lesions, poor detection of early dysplastic changes, and too short follow-up periods in prospective studies. The latest systematic review assessed an overall transformation rate of 1.40% [11], but this was probably underestimated if one takes into account the previously discussed limitations. Nowadays, the WHO and some authors consider lichenoid reactions to have a malignant potential [3,12]. However, the exact transformation rate is unknown since diagnostic criteria are not always clear-cut. Further studies are necessary to elucidate the true premalignant role of such lesions.

## 4. Conclusions

The purpose of this article is to report an unusual case of drug-induced reaction of the oral mucosa, induced by allopurinol, for which a complete healing was obtained after withdrawal of the drug. Clinicians must be aware of the recognition and management of oral lichenoid drug reactions. The diagnosis may be difficult, since a great overlap in clinical and histopathological presentation with oral idiopathic lichen planus exists. Although a matter of controversy, diagnosis and follow-up of these lichenoid lesions seems all the more important due to potential malignant transformation.

## Figures and Tables

**Table 1:** Clinical and histopathological characteristics of allopurinol-induced oral lesions in patients reported in 3 publications.

		Clinical Features	Allopurinol Indication	Histopathological Features	Outcomes
Chau et al. 1984 [7]	M, Caucasian, 53 y	Ulcerations Keratotic striae and plaques	Gout	No biopsy	Healing of ulcers Persistence of the keratosis
	M, Caucasian, 56 y	Ulcerations on the lip, buccal mucosa and tongue	Gout	No biopsy	Healing of ulcers Persistence of the keratosis
	M, Caucasian, 60 y	Ulcerations Keratotic striae on the lip, buccal mucosa and tongue	Gout	LP versus lichenoid reaction	Persistence of minor ulcerations
Nair et al. 2005 [8]	F, Chinese, 75 y	Erosions and ulcerations Keratotic striae and plaques on the buccal mucosa and tongue	Gout	No biopsy	Resolution of erosions Persistence of faint keratosis
Present case	M, Caucasian, 59 y	Buccal ulceration Keratotic striae on the lip, buccal mucosa and tongue	Gout	LP versus lichenoid reaction	Complete healing
